# Finer leaf resolution and steeper beam edges using a virtual isocentre in concurrence to PTV-shaped collimators in standard distance – a planning study

**DOI:** 10.1186/s13014-017-0826-8

**Published:** 2017-05-25

**Authors:** Klaus Bratengeier, Barbara Herzog, Sonja Wegener, Kostyantyn Holubyev

**Affiliations:** 1 Department of Radiation Oncology, University of Würzburg, Josef-Schneider-Str. 11, 97080 Würzburg, Germany; 2 Martin-Luther-Universität Halle-Wittenberg, Institute of Physics, Von-Danckelmann-Platz 3, 06120 Halle (Saale), Germany; 3 University of Freiburg, Klinik für Strahlenheilkunde, Abt. Medizinische Physik, Robert-Koch-Str. 3, 79106 Freiburg, Germany

**Keywords:** Radiotherapy, Stereotactic Irradiation, Robotic table motion, Multi-leaf collimator, Planning study, Virtual isocentre

## Abstract

**Purpose:**

Investigation of a reduced source to target distance to improve organ at risk sparing during stereotactic irradiation (STX).

**Methods:**

The authors present a planning study with perfectly target-volume adapted collimator compared with multi-leaf collimator (MLC) at reduced source to virtual isocentre distance (*SVID*) in contrast to normal source to isocentre distance (*SID*) for stereotactic applications. The role of MLC leaf width and 20–80% penumbra was examined concerning the healthy tissue sparing. Several prescription schemes and target diameters are considered.

**Results:**

Paddick’s gradient index (GI) as well as comparison of the mean doses to spherical shells at several distances to the target is evaluated. Both emphasize the same results: the healthy tissue sparing in the high dose area around the planning target volume (PTV) is improved at reduced *SVID* ≤ 70 cm. The effect can be attributed more to steeper penumbra than to finer leaf resolution. Comparing circular collimators at different *SVID* just as MLC-shaped collimators, always the GI was reduced. Even MLC-shaped collimator at *SVID* 70 cm had better healthy tissue sparing than an optimal shaped circular collimator at *SID* 100 cm.

Regarding penumbra changes due to varying *SVID*, the results of the planning study are underlined by film dosimetry measurements with Agility™ MLC.

**Conclusion:**

Penumbra requires more attention in comparing studies, especially studies using different planning systems. Reduced *SVID* probably allows usage of conventional MLC for STX-like irradiations.

**Electronic supplementary material:**

The online version of this article (doi:10.1186/s13014-017-0826-8) contains supplementary material, which is available to authorized users.

## Introduction

### General aspects

Normal linacs are not considered to be suitable for modern stereotactic irradiation (STX). The main reason is the isocentric leaf width of common multi-leaf collimators (MLC). Typical isocentric 0.5 cm MLCs are assumed too coarse for STX. Leaf widths of the order of 0.3 cm are recommended by Bortfeld et al. [1]. However, this value is subject to the typical 20–80% penumbra width of 0.25 cm to 0.3 cm. Since then, several authors more often consider MLC types and leaf widths [2–7], whereas other parameters which determine the dose gradient, such as the penumbra, are rarely examined or even varied. For example, the authors refer the reader to the method of penumbra control using intermediate energy photons [8] proposed by O’Malley et al. [8] or small-field flattening filters proposed by S.J. Thomas [9] in an older work,.

### Virtual isocentre

The purpose of the present work is to investigate whether a reduced source to target distance combined with MLC (0.5 cm isocentric leaf width) can compete against reference source to isocentre distance (*SID*) 100 cm combined with an ideal round collimator (a limit of infinitely narrow leaves). The circular collimator is the ideal shape for spherical targets only. The reduced source to target distance can be realized using, e. g., a virtual isocentre approach (Bratengeier K, Holubyev K, Wegener S: Distance-dependent penumbra: Theoretical considerations and practical implications for stereotactic irradiation using a MLC, submitted): Table positions are a function of the gantry angle and the isocentric table angle in a way that a selected target point is always hit by the beam central axis in a certain fixed distance from the source. This point behaves like an isocentre and is therefore called “virtual isocentre”. Its distance from the source is called source to virtual isocentre distance (*SVID*) which is allowed to differ from *SID* 100 cm. The apparatus as a whole behaves like a linac with changed *SID*, but however, the radiation head remains unchanged. For the present study we consider the *SVID* reduced to 70 cm, which is a reasonable distance for head applications, and a *SVID* of 50 cm to demonstrate the trends at further reduced *SVID*. Our special interest is to examine the role of reduced MLC leaf width separately from penumbra effects regarding improved healthy tissue sparing at reduced *SVID*.

## Methods

### Planning system and target definition

The planning study was performed by means of the therapy planning system (TPS) Philips Pinnacle^3^™ version 9.10. A virtual sphere of diameter 20 cm and physical density 1.0 g/cm^3^ was created in TPS on a 0.1 cm sliced CT. The spherical planning target volume (PTV) was placed in the centre. PTV of diameters (∅) of 1.0 cm, 1.3 cm and 1.7 cm were created inflating a nearly point size central object using Pinnacle^3^™ expansion functions. Using three different target diameters was intended to investigate the effect of different curvatures. The diameters were chosen small to recognize the effects of MLC induced grating.

### Evaluation parameters

#### Mean dose to spherical shells

To ensure technique-independent characterisation of the dose distribution, concentric spherical shells with borders at 0.3, 0.7, 1.0, 1.3, 1.6, 1.9, 2.2, 2.5, 2.8, 3.0, 4.0, 6.0 10.0 and 20.0 cm were created in the TPS. In the limit of isotropic irradiation, the mean dose at each shell was identical with the dose to an infinitely thin shell of an effective radius *r* dividing the original shell into two parts of equal volume. The mean dose to the shells was read off in TPS and plotted against the effective radius. In principle, a single beam would be sufficient for the calculation, as mean doses are additive. But a single beam is subject to direction dependent inaccuracies of voxel and slice based planning system and of accidental anisotropies. Therefore, a quasi-isotropic arrangement of sixteen beams was chosen [10] which was equivalent to multi-arc techniques for doses between 100 and 12% of the maximum dose, as illustrated in Additional file 1.

#### Conformity index and gradient index

For further evaluations, Paddick’s conformity index PCI [11] and gradient index GI [12] were chosen:

The *PCI* was defined as

$$ P C I={\left(\frac{ V ol{\left(\mathrm{PTV}\cap \mathrm{TV}\right)}^2}{PTV* TV}\right)}_{ICRU}={\left(\frac{TT{ V}^2}{PTV* PIV}\right)}_{Neurosurg}, $$

where TV denoted the “treated volume” according to ICRU 83 [13], *TV* = Vol(TV) was the volume of this structure. PTV represented the planning target volume to be covered with the desired prescription dose; PTV $\cap$ TV was the intersection of PTV and TV. In the nomenclature of Neurosurgery “TV” is often used as target volume [14]; to avoid confusion with treated volume, here “PTV” was used instead. *TTV* was the treated target volume and *PIV* stood for the planning isodose volume (= treated volume according ICRU 83 = Reference-isodose enclosed volume).

Paddick’s gradient index *GI* was defined as the ratio between the volume enclosed by the half of the reference dose *PIV*_*0.5*_ and *PIV*:

$$ G I=\frac{PI{ V}_{0.5}}{ PI V}\ . $$

A *GI* in stereotactic applications was aimed to be below 3.0 [12].

#### V_66.7%_

Additionally, the volume in which the dose exceeds 66.7% of the prescription dose, *V*_*66.7%*_ *,* was evaluated, known as “V12” for 18 Gy prescription in literature. However, within the study the dose to the isocentre was set to the maximum dose of 10 Gy. This choice does not restrict the generality of any results, as the scaling remains free. The prescription was set to the 70%-, 80%- and 90%-isodoses (these situations are called “D70%”, “D80%”, “D90%”); this means 7 Gy, 8 Gy or 9 Gy, respectively, were aimed to surround the PTV exactly in a way described below.

### Planning details

The MLC leaves were positioned using the “Expose-PTV” block margin function of the Pinnacle^3^ planning system in such a way that the MLC leaves touched the margin around the PTV in beam eye view projection. Also beams with PTV conformal, nearly circular blocks were created using the margin. The same margin was used for all beams of a given technique. To study the influence of prescription, the 70%-, 80%- and 90%-doses of the reference isocentre dose (=100%) were chosen as “PTV-surrounding”, respectively. They cover at least 99% of the *PTV*. A block thickness was chosen to produce the same transmission as the standard Elekta Agility^TM^ MLC with 0.5 cm leaf width at *SID* 100 cm. To carve out the impact of *SVID* and consequential effects of the penumbra changes, same conditions for block and MLC were simulated; the virtual block was placed in the MLC distance. Thus, the block behaved like a MLC with infinitely narrow leaves. To compare the techniques with each other, the same dose was prescribed to the isocentre; the PTV margin was iteratively adapted to achieve the same mean dose inside the PTV. Details of this method are described in Additional file 2. Changes of the penumbra were simulated exemplarily by a superposition of beams with different diameters. Beam charact eristics (profiles) were determined according to Additional file 3.

## Results

The authors considered deviances of ± 2% of *PCI* as “nearly equal”; effects of similar order could occur due to dose grid effects and effects of digitization (voxel generation) of a spherical object (see [15] for volume effects of structure definition). Thus, only for a PTV diameter of 1.0 cm and a *SID* of 100 cm, the *PCI* differed markedly for MLC compared with circular collimator (Table 1).

**Table 1:** Paddick’s conformity index PCI for investigated constellations

*PCI*			Circular Collimator			MLC 0.5 cm (isocentric)		
		*SID or SVID* [cm]	100	70	50	100	70	50
∅ [cm]	Prescription							
1.0	D80%		0.87	0.92	0.92	0.79	0.88	0.94
1.3	D70%		0.88	0.92	0.93	0.85	0.89	0.93
1.3	D80%		0.87	0.89	0.90	0.82	0.88	0.90
1.3	D90%		0.85	0.83	0.84	0.85	0.86	0.86
1.7	D80%		0.91	0.92	0.93	0.85	0.92	0.92

Reduced *SVID* influenced both, penumbra and effective leaf width. The following sections were conceived to separate penumbra and lead width effects.

### *SID* 100 vs. *SVID* 70/50

The *GI* (Table 2) always decreased for reduced *SVID*, for all target diameters and prescriptions. It fell below the critical value of 3.0 for D70%, ∅ 1.3 cm and D80%, ∅ 1.7 cm.

**Table 2:** Gradient index GI for investigated constellations

*GI*			Circular Collimator			MLC 0.5 cm (isocentric)		
		*SID or SVID* [cm]	100	70	50	100	70	50
∅ [cm]	Prescription							
1.0	D80%		3.90	3.45	3.20	4.21	3.74	3.63
1.3	D70%		2.91	2.70	2.61	3.15	2.92	2.77
1.3	D80%		3.26	2.83	2.78	3.57	3.22	3.01
1.3	D90%		4.19	3.69	2.44	4.65	4.05	3.73
1.7	D80%		2.86	2.63	2.52	3.15	2.85	2.69

**Fig. 1:**
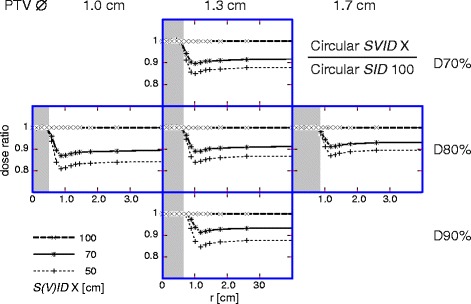
Circular collimator: *SVID* dependence of radial dose. Radial dose distribution for circular collimator at *SVID* 70 cm (continuous) and at 50 cm (*thin dashed*), normalized to the distribution for circular collimator at *SID* 100 cm. *Shaded* area: PTV. *Left*: PTV ∅ 1.0 cm; *middle*: PTV ∅ 1.3 cm; *right*: PTV ∅ 1.7 cm. Prescriptions: *top*: D70%; *middle*: D80%; *bottom*: D90%. *S(V)ID*: *SID* or *SVID*

**Fig. 2:**
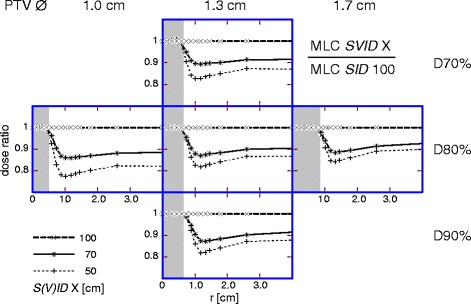
MLC: *SVID* dependence of radial dose. Radial dose distribution for MLC at *SVID* 70 cm (continuous) and 50 cm (*thin dashed*), normalized to the distribution for MLC at *SID* 100 cm. Shaded area: PTV. *Left*: PTV ∅ 1.0 cm; *middle*: PTV ∅ 1.3 cm; *right*: PTV ∅ 1.7 cm. Prescriptions: *top*: D70%; *middle*: D80%; *bottom*: D90%. *S(V)ID*: *SID* or *SVID*

In more detail, the effect of reduced *SVID* on a radial dose distribution is shown Figs. 1 and 2 for circular collimator and MLC, respectively. The distributions were normalized to that one at *SID* 100 cm. For both, circular collimator and MLC, the decrease of the dose to the surrounding healthy tissue at the reduced *SVID* was almost independent from the prescription. However, the effect of healthy tissue sparing utilizing reduced *SVID* was more pronounced for the “MLC” situation (Fig. 2); most probably due to the additional effect of finer approximations of a circular beam formed by thinner MLC leaves. The next section is intended to quantify the MLC and penumbra contributions.

### Circular collimator vs. MLC

**Fig. 3:**
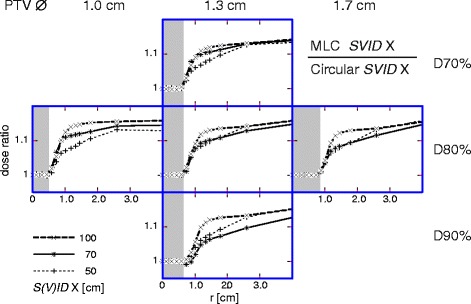
MLC compared with circular collimator at same *SVID.* Radial dose distribution for MLC normalized to the distribution for circular collimator at different *SID*: 100 cm (*thick dashed*), *SVID* 70 cm (continuous), and 50 cm (*thin dashed*). *Shaded* area: PTV. *Left*: PTV ∅ 1.0 cm; *middle*: PTV ∅ 1.3 cm; *right*: PTV ∅ 1.7 cm. Prescriptions: *top*: D70%; *middle*: D80%; *bottom*: D90%. *S(V)ID*: *SID* or *SVID*

The gradient index in Table 2 clearly showed lower *GI* values for circular collimators with respect to MLC. Accordingly, Fig. 3 compares the dose distributions for a MLC and a circular collimator at the same *SVID*. Clearly, the MLC always led to an additional dose in the surrounding healthy tissue. This additional contribution decreased at reduced *SVID*. This decrease was not always most effective for *SVID* 50, assumedly due to”digitization” effects of the circular shape by slots of finite width. Obviously, the influence of MLC-beam-shaping was less than the distance effects shown in Fig. 1.

### MLC at SVID 70/50 or optimal shape at SID 100?

**Fig. 4:**
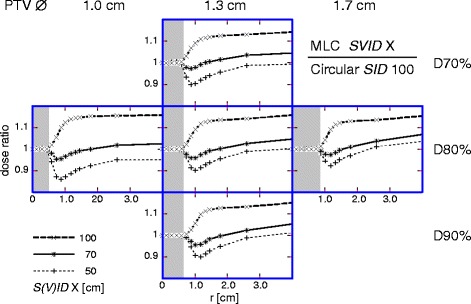
MLC at various *SVID* compared with circular reference collimator at *SID* 100 cm. Radial dose distribution for MLC at different *SVID* normalized to the distribution for the reference technique with circular collimator at *SID* 100 cm: MLC at *SID* 100 cm (*thick dashed*), *SVID* 70 cm (continuous), and 50 cm (*thin dashed*). *Shaded* area: PTV. *Left*: PTV ∅ 1.0 cm; *middle*: PTV ∅ 1.3 cm; *right*: PTV ∅ 1.7 cm. Prescriptions: *top*: D70%; *middle*: D80%; *bottom*: D90%. *S(V)ID*: *SID* or *SVID*

Can a reduced *SVID* compensate for the “roughness” of MLC beam shaping? In four of the five cases listed in Table 2, the *GI* for the MLC at *SVID* 70 cm was lower than ideal beam shaping by a circular collimator at *SID* 100 cm (and in the one remaining case, the values differed only marginally). Clearly, the steeper penumbra at reduced *SVID* overcompensated the deteriorating effect of MLC beam shaping even for the 0.5 cm MLC. In more detail, the cumulative effect of combining reduced *SVID* with MLC can be deduced from Fig. 4, which shows the MLC dose distributions normalized to that of circular collimator at standard *SID* 100 cm. In any case for the high dose region (hence, up to double PTV radius), using a MLC at *SVID* 70 cm was preferable to a perfect beam shaper (using appropriate circular collimators of optimized diameter) at *SID* 100 cm.

### Clinical relevance?

All results from sections A, B, C were also reflected in Table 3. For a MLC, V_66.7%_ was reduced from 13% up to 17%, if *SID* 100 cm was reduced to *SVID* 70 cm. For ∅ 1.0 cm this means a reduction of more than half of the target volume; even for ∅ 1.7 cm it resulted in a reduction of one third. If a circular collimator at *SID* 100 cm was replaced by a MLC at *SVID* 70 cm, V_66.7%_ was still reduced by 11 ± 4%. Aspects of the clinical relevance of these results were also shown by an example in Additional file 4.

**Table 3:** Volume of 66.7% (V_66.7%_) of prescription dose for investigated constellations

V_66.7%_			Circular Collimator			MLC 0.5 cm (isocentric)		
		*SID or SVID* [cm]	100	70	50	100	70	50
∅ [cm]	Prescription							
1.0	D80%		1.87	1.58	1.44	2.09	1.73	1.50
1.3	D70%		3.01	2.62	2.44	3.30	2.87	2.57
1.3	D80%		3.58	3.12	2.89	3.98	3.34	3.11
1.3	D90%		4.62	4.22	3.88	5.06	4.28	4.00
1.7	D80%		6.34	5.72	5.38	7.05	6.08	5.67

### Effects of superposition and penumbra increase

To evaluate the increase in the healthy tissue dose resulting from an enlarged penumbra for combined beam arrangements, we artificially raised the penumbra at constant *SID* using a superposition of beams with differing diameters.

**Fig. 5:**
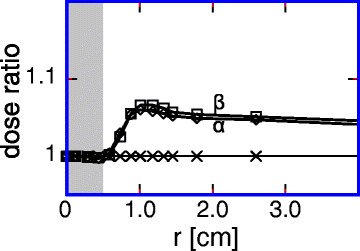
Simulation of enlarged penumbra. Radial dose distribution for superimposed beam arrangements normalized to reference beam arrangement generated using PTV margin. *Shaded* area: PTV. *Crosses*: Reference (PTV ∅ = 1.0 cm, D80%, circular collimator, PTV margin 0.00 cm). *Diamonds*: superposition α (generated from PTV margins additionally −0.10 cm and +0.10 cm, respectively). *Squares*: superposition β (generated from PTV margins additionally +0.15 cm and −0.05 cm, respectively). See text for explanation

As a reference, we considered a PTV ∅ of 1.0 cm and used circular collimator with a margin around PTV to meet the demands of a D80% prescription (Fig. 5). We considered superposition of pairwise wider and narrower beams, identical for the whole arrangement. Two cases were generated with PTV margins of additional +0.10 cm and −0.10 cm (case α), and +0.15 cm and −0.05 cm (case β). The same prescription was used for both cases. The relative beam weights were adjusted to achieve the same mean dose to the PTV (71.5 and 28.5%, respectively, for α, 35.4 and 64.6% for β). We found that for 0.20 cm difference between PTV margins the penumbra increased from 0.35 cm (reference) to 0.37 cm (α) or 0.38 cm (β). The associated increase of the healthy tissue dose reached its maximum of about 7% at a distance of 0.5 cm from the PTV, for both α and β, see Fig. 5. The penumbra increase depended on the difference between margins only. Assuming this dependence to be linear, we estimated the systematic increase of the healthy tissue dose at *SID* 100 cm for a margin step 0.02 cm not to exceed 0.7%. At *SVID* 70 cm for margin step 0.04 cm it did not exceed 1.4%, and at *SVID* 50 cm for margin step 0.10 cm 3.5%. Thus, combining beams to achieve the prescribed radial mean PTV dose (see Additional file 1 and methods section) did not influence the results for the healthy tissue at *SID* 100 cm and *SVID* 70 cm. For *SVID* 50 cm, used for demonstration purposes, the systematic dose increase from beam superposition did not exceed 3.5%.

## Discussion

### MLC beam shaping

**Fig. 6:**
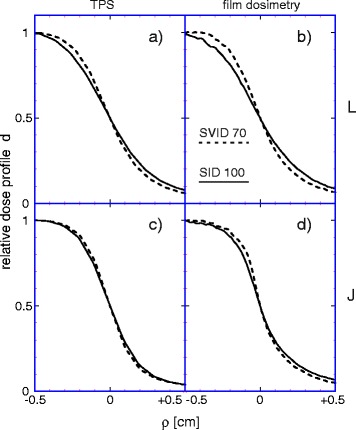
Measured and calculated beam edges. Source to isocentre distance (*SID*) 100 cm (*continuous line*) and source to virtual isocentre distance (*SVID*) 70 cm (*dashed line*): Beam edges are set to 50% point for beams of effective 10 × 10 mm^2^ (nominally 10 × 10 mm^2^ for *SID* 100 cm, 14 × 14 mm^2^ for *SVID* 70 cm, respectively). *Left* side (a + c): planned in TPS. *Right* side (b + d): film measurements. Upper row L (a + b): leaf direction; lower row J (c + d): jaw direction. Depth 10 cm, central axis dose is normalized to 1.0. See also Additional file 3.

Not surprisingly, the use of a MLC instead of optimal circular collimators leads to more dose to the healthy tissue around the PTV, see Fig. 3. However, the effect of MLC is less pronounced than intuitively expected. At *SID* 100 cm the MLC provides coarser approximation of the circular collimator as at *SVID* 70 cm or even 50 cm. In the worst case of 1.0 cm diameter spherical PTV, a 1.0 cm × 1.0 cm square MLC approximation leaves 27% of the area around the PTV unblocked, if the two MLC leaves, at both sides each, touch the PTV in the beam projection. For *SVID* 50 cm the unblocked area would numerically be 19% for four leaves at both sided of a circle. Interestingly, the planning study shows that the effect of MLC vs circular collimator (Fig. 3) increases the dose to the healthy tissue only by half of that, 10–13%. This is due to the fact that, depending on prescription, the MLC apertures have to be generated from negative PTV margins [16] and thus not always fully contribute to the primary dose in healthy tissue. In contrast, the penumbra in any case contributes to the primary dose in the healthy tissue, so its effect seems to be always larger. The deterioration of the dose falloff from MLC apertures can be overcompensated by a reduced *SVID*, as demonstrated in Fig. 4. In the high dose area adjacent to the PTV, the *SVID* ≤ 70 cm combined with MLC performs better than the perfectly adapted circular collimator at standard *SID* 100 cm. The effect must be traced back to the steeper penumbra as depicted in Fig. 6 and quantified in Fig. 5. An increase of 20–80% penumbra width by 0.02 or 0.03 cm leads to 7% larger healthy tissue dose. For the MLC as modelled in the planning system *SID* 100 cm compared to *SVID* 70 cm leads to penumbra increase of 0.03 cm in J direction, 0.08 cm in L direction. This is semiquantitatively in good agreement with the increase of the healthy tissue dose of about 12–14% seen in Fig. 2 (D80%, ∅ 1.3 cm).

### Validity of the planning study results

Note that the conclusions above probably are quite general and independent from the beam shaping device, although a special (spherical) PTV shape was chosen. The present study was performed for small spherical targets of different diameters. These targets stand for objects with various curvatures. The simple circular shape allows the definition of an optimal circular aperture, which is regarded as the limit of an infinitely small leaf width. All results were qualitatively independent from the PTV diameter. They were also independent from different prescription schemes, be that D70%, D80% or D90%.

### The role of penumbra steepness

For circular collimator (Fig. 1) combined with reduced *SVID*, the improved healthy tissue sparing comes from the penumbra decrease. For MLC, the improvement in healthy tissue sparing at reduced *SVID* (Fig. 2) is dominated by the penumbra decrease, which can be inferred by comparing Fig. 2 vs. Fig. 1 and can be concluded from Fig. 4. As long as penumbra is decreased by 0.02 cm or more at reduced *SVID*, the MLC performs better than an ideal collimator at standard *SID* 100 cm. Although this was shown for the Agility^TM^ head, this result can be assumed relevant also for other MLCs, as the effect can be traced back to the geometrical penumbra. Independent from the present planning study, the importance of the penumbra can be deduced from more theoretical considerations (Bratengeier K, Holubyev K, Wegener S: Distance-dependent penumbra: Theoretical considerations and practical implications for stereotactic irradiation using a MLC, submitted).

In this study, the healthy tissue sparing effect of reduced *SVID* decreases for a larger PTV radius independently of beam shaping device, see Figs. 1 and 2. The sparing effect is dominated by penumbra decrease, which becomes less important for larger PTV: the volume of a thin layer around PTV, where penumbra dose dominates, becomes smaller relative to the PTV volume itself.

### Advantages of reduced SVID

The effect of using MLC instead of ideal circular collimator (Fig. 3) is found (almost independent of *SVID* and prescription) at the level of 10% dose increase to the healthy tissue. The effect of reduced *SVID* is proven for *SVID* 70 cm, practical for head irradiations, at the level of 10% dose decrease due to decreased penumbra. Thus, at *SVID* 70 cm combined with MLC the two effects compensate and the plan quality is at least as good as at *SID* 100 cm combined with circular collimator (Fig. 4). In fact, the additional improved beam shaping due to narrower effective leaf widths ensures even further healthy tissue sparing.

### Relation to neurosurgical literature data

The increase of quality by using *SVID* 70 cm or less is clearly significant and relevant: Dose to the surrounding decreases, as can be seen from *GI* and *V*_*66.7%*_.

The *GI* for higher dose maxima and for more extended targets decreases below the intended *GI* <3.0 as demanded by the radiosurgery consortium [14], even for the 0.5 cm MLC at *SVID* 70 and below. For targets of 1.0 cm diameter, D50% should be used instead of D70%, because Paddick and Lippitz demonstrated a strong decrease of *GI* with increasing maximum dose [12] for typical beam profiles.

The presented results even assume infinitely variable collimator diameters, which will not be available for fixed sets of applicators of different diameters. In contrast, MLC and jaw can be steered in sub-millimeter range, as in the presented study. This fact additionally favours the MLC-*SVID*-approach. This is all the more the case for the shaping of non-spherical targets. Certainly, shaping by a MLC will be even more advantageous compared to circular applicators, if non-spherical targets are to be treated: the application time may be reduced. The treatment time is further decreased as a result of the inverse-quadratic law.

Recently, several authors pushed dynamic techniques using table movement [17] or complex leaf steering (see i.e., [18]) that require fail-safe and precise table and leaf movements, much more than needed for the present study. Therefore we excluded safety and precision aspects for the present study.

## Conclusion

Using reduced *SVID* (e.g., in form of virtual isocentre) in combination with beam shaping device of any kind reduces the penumbra. Decreased penumbra provides an important contribution to the sparing of surrounding healthy tissue. The penumbra decrease obviously accounts for better healthy tissue sparing at reduced *SVID*. The present work proves that this effect dominates the decrease of healthy tissue dose at reduced *SVID* combined with MLC. In summary, for an Elekta Agility™ head, even a 0.5 cm leaf MLC at *SVID* 70 cm distance allows at least as good or even better healthy tissue sparing as an optimally shaped collimator at *SID* 100 cm.

In summary, using the *SVID* in reduced distance mode, the MLC-techniques could deliver approximately equal or better plan quality due to overcompensation of MLC “roughness” by the reduced penumbra.

The authors are convinced that the penumbra requires more attention in comparing studies, especially studies using different planning systems. This view is supported by theoretical work presently considered for publishing (Bratengeier K, Holubyev K, Wegener S: Distance-dependent penumbra: Theoretical considerations and practical implications for stereotactic irradiation using a MLC, submitted). Guidelines for stereotactic irradiation should not only define the leaf width but should also contain requirements for the penumbra [19].

Further studies should address more complex patient related targets and other types of linacs or sources combined with a virtual isocentre and its variable source-to-patient distances.

## Additional files

Additional file 1: Relevance of 16 beam quasi-isotropic irradiation [10]. (PDF 10 kb)

Additional file 2: Details of beam fine-tuning [20]. (PDF 47 kb)

Additional file 3: Planning system and real beams [21]. (PDF 12 kb)

Additional file 4: Clinical example [12]. (PDF 8 kb)
