# Supplementary material for: Finer leaf resolution and steeper beam edges using a virtual isocentre in concurrence to PTV-shaped collimators in standard distance – a planning study
Source: Radiat Oncol. 2017 May 25;12:88. doi: 10.1186/s13014-017-0826-8 (PMC5445413; doi:10.1186/s13014-017-0826-8)
Supplement: Supplementary file 1 — Relevance of 16 beam quasi-isotropic irradiation [10]. (PDF 10 kb) [file 13014_2017_826_MOESM1_ESM.pdf]

ADDITIONAL FILE 1:

RELEVANCE OF 16 BEAM QUASI-ISOTROPIC IRRADIATION

The quasi-isotropic 16 beam technique as described by Bratengeier et al.[10] was compared with 1 up to 9 arc techniques to evaluate its relevance for generalized, reliable statements. Table 4 compiles the results for the PTV diameter 1.3 cm (V_{PTV} = 1.15 cm^3). To distribute the beams evenly, the beam directions were chosen using platonic solids, the dodecahedron and the icosahedron, according to Bratengeier et al.[10]. Thus even distribution over 2 Pi was guaranteed (as long as entrances and exits are regarded as equivalent, even 4 Pi). All relevant isodose-included volumes were comparable to the multiple arc techniques. The dose distribution was nearly identical to the dose distribution by multiple arc techniques; it could be best compared with a 5 arc technique as can be deduced from table A1.

Table A1:

Comparison of plan quality parameters of quasi-isotropic 16 beam technique and arcs.

	1 Arc	2 Arcs	3 Arcs	5 Arcs	9 Arcs	**16 Beams quasi-isotropic**
**PTV**						
D_{max} (PTV)	10.0	10.0	10.0	10.0	10.0	**10.0**
D_{mean} (PTV)	9.23	9.30	9.31	9.30	9.29	**9.29**
D_{min} (PTV)	7.22	7.85	7.91	7.85	7.91	**7.87**
**PTV/OAR**						
CI	0.87	0.83	0.86	0.85	0.86	**0.87**
**OAR**						
V_{8Gy}	1.70	1.77	1.74	1.74	1.73	**1.72**
$V_{67\%}$	4.21	3.61	3.61	3.61	3.58	**3.57**
V_{4Gy}	7.71	5.78	5.69	5.59	5.63	**5.64**
V_{2Gy}	36.0	18.5	14.3	14.2	14.0	**14.0**
GI	4.54	3.27	3.27	3.22	3.25	**3.28**
D_{max} (r 1.3 cm)	8.43	9.05	8.81	8.77	8.86	**8.73**
D_{max} (r 1.8 cm)	6.43	6.12	5.97	5.94	5.88	**5.99**
D_{max} (r 3.0 cm)	3.65	3.11	2.29	2.27	2.15	**2.20**
D_{max} (r 6.0 cm)	2.01	1.64	1.11	0.86	0.66	**0.90**
D_{mean} (r 1.6-1.3 cm)	7.35	7.54	7.52	7.50	7.49	**7.47**
D_{mean} (r 2.2-1.8 cm)	4.34	4.40	4.37	4.36	4.36	**4.34**
D_{mean} (r 4.0-3.0 cm)	1.61	1.32	1.31	1.31	1.31	**1.30**
D_{mean} (r 10.0-6.0 cm)	0.67	0.26	0.26	0.26	0.26	**0.26**

Irradiation of a sphere of 1.3 cm diameter.

Prescription dose D_{ref} = 8.0 Gy (D80%);

1 Arc: 181° > 179°; x Arcs: 30° > 120° or 210° > 330°, each;

table angles equidistant;

16 beams: quasi-isotropic according to Bratengeier et al. [10].

“D_{max} (r 1.3 cm)” means maximum dose in a distance of 1.3 cm.

“D_{mean} (r 1.6-1.3 mm)” is the mean dose in a spherical shell concentric around the PTV with inner radius 1.3 cm and outer radius 1.6 cm.
