# Supplementary material for: Finer leaf resolution and steeper beam edges using a virtual isocentre in concurrence to PTV-shaped collimators in standard distance – a planning study
Source: Radiat Oncol. 2017 May 25;12:88. doi: 10.1186/s13014-017-0826-8 (PMC5445413; doi:10.1186/s13014-017-0826-8)
Supplement: Supplementary file 2 — Details of beam fine-tuning [20]. (PDF 47 kb) [file 13014_2017_826_MOESM2_ESM.pdf]

ADDITIONAL FILE 2:

DETAILS OF BEAM FINE-TUNING

The PTV block margin could be changed in Pinnacle only stepwise, in 0.02 cm steps for *SID* 100 cm, 0.04 cm for *SVID* 70 cm, and 0.10 cm for *SVID* 50 cm, which allowed achieving a prescribed PTV mean dose to better then 1.5 %. By superposing two beam arrangements generated from both nearest allowed PTV block margins, a prescribed PTV mean dose deviation of $\leq$ 0.1 % could be attained by adjusting the weights of the arrangements.

The superposition of beam arrangements insignificantly increased the penumbra and thus the dose load to the healthy tissue. We investigated the associated healthy tissue dose load[20] and found that this effect did not exceed 2% at any point of the radial dose distribution for practical *SID* 100 cm and *SVID* 70 cm and, therefore, it can be neglected.
