# Supplementary material for: Finer leaf resolution and steeper beam edges using a virtual isocentre in concurrence to PTV-shaped collimators in standard distance – a planning study
Source: Radiat Oncol. 2017 May 25;12:88. doi: 10.1186/s13014-017-0826-8 (PMC5445413; doi:10.1186/s13014-017-0826-8)
Supplement: Supplementary file 3 — Planning system and real beams [21]. (PDF 12 kb) [file 13014_2017_826_MOESM3_ESM.pdf]

ADDITIONAL FILE 3:

PLANNING SYSTEM AND REAL BEAMS

The planned dose profiles for the MLC were compared with the measurement. The circular collimators were virtual ones; therefore they were not available for experiments. Measurements were performed by film dosimetry for *SVID* 70 cm with a quadratic MLC-shaped beam of nominal 1.4 cm by 1.4 cm (approx. 1.0 cm by 1.0 cm at *SVID* 70 cm) and *SID* 100 with 1.0 cm by 1.0 cm. An Elekta Linac with 6 MV, flatness filter and Agility™ head with rounded leaves 0.5 cm wide at isocentre (SID 100 cm) was commissioned in Pinnacle^{3}™. The films were placed under 10 cm of solid water. After irradiation they were scanned on an Epson Expression XL11000 film scanner, a Gafchromic EBT3 (Ashland, USA) was utilized, (see Micke et al.[21]). All films were centrally placed under glass slab for compression and scanned with 150 dpi (48 bit RGB) without colour corrections. The same film orientation was always used. The scans were digitally stored for evaluation in lossless .tif format. In Fig 6, beam edges are depicted for comparison. All edges were shifted until half height relative dose was at $\rho = 0$ mm. On the left, (a) and (c), the planned dose for two *SVID* 70 cm and *SID* 100 cm is shown, on the right, (b) and (d), the corresponding measured dose is shown. The measured dose is the mean over two independent measurements at both sides of the beam. The 20%-80% penumbra at *SID* 100 cm was always larger than at *SVID* 70 cm. Mean penumbra over four single measurements was 0.25 cm (3.4 mm) at *SVID* 70 cm and 0.31 cm (0.44 cm) at *SID* 100 cm in jaw direction (leaf direction, respectively). For the planned dose, the mean penumbra over two sides of the beam was 0.27 cm (0.35 cm) at *SVID* 70 cm and 0.30 cm (0.43 cm) at *SID* 100 cm in jaw direction (leaf direction, respectively). Real beams were slightly steeper than their representation in the planning system; however the deviations were similar for *SID* 100 and *SVID* 70 and should compensate each other in a comparison.
