# Supplementary material for: Finer leaf resolution and steeper beam edges using a virtual isocentre in concurrence to PTV-shaped collimators in standard distance – a planning study
Source: Radiat Oncol. 2017 May 25;12:88. doi: 10.1186/s13014-017-0826-8 (PMC5445413; doi:10.1186/s13014-017-0826-8)
Supplement: Supplementary file 4 — Clinical example [12]. (PDF 8 kb) [file 13014_2017_826_MOESM4_ESM.pdf]

ADDITIONAL FILE 4:
CLINICAL EXAMPLE

One example should be presented to emphasize the relevance of the results. More examples should be reserved to a more extended planning study based on clinical cases.

A spindle shaped PTV of main axes 1.4 cm, 1.0 cm and 1.0 cm and 0.75 cm^3 volume enclosed a gross tumour volume (GTV) of 0.33 cm^3. A maximum dose 12.5 Gy and a PTV-"surrounding" prescription dose of 10.0 Gy (100%) were demanded. MLC plans at *SID* 100 and *SVID* 70 were compared, two versions each: D100% = 10 Gy and D99% = 10 Gy.

Table A4:

	D100%		D99%	
	SID 100 cm	*SVID* 70 cm	*SID* 100 cm	*SVID* 70 cm
PTV D_{mean} [Gy]	11.7	11.7	11.6	11.6
PTV D_{min} [Gy]	10.1	10.1	9.7	9.7
GTV D_{mean} [Gy]	12.1	12.1	12.0	12.1
GTV D_{min} [Gy]	11.1	11.2	10.9	11.0
PCI	0.746	0.751	0.790	0.777
Brain-PTV D_{mean} [Gy]	0.240	0.222	0.233	0.215
$V_{66.7\%}$ [cm^3]	2.61	2.44	2.46	2.32
GI	3.90	3.61	3.97	3.64

For *SVID* 70 cm the $V_{66.7\%}$ -volume was always 0.14 up to 0.17 cm^3 smaller than for *SID* 100 cm, which is one half of the GTV volume (or 6% of the $V_{66.7\%}$). Additionally the mean dose to the healthy brain was about 10% lower. *GI* was also reduced. It should be noted that Paddick et al.[12] achieved a *GI* between 3.2 and 4.2 for target diameters between 0.8 cm and 1.4 cm at a D80% prescription as used here.
